# Single‐Step Genome‐Wide Association Study of Factors for Evaluated and Linearly Scored Traits in Swedish Warmblood Horses

**DOI:** 10.1111/jbg.12923

**Published:** 2025-01-04

**Authors:** Anahit Nazari‐Ghadikolaei, W. Freddy Fikse, Åsa Gelinder Viklund, Sofia Mikko, Susanne Eriksson

**Affiliations:** ^1^ Department of Animal Biosciences Swedish University of Agricultural Sciences Uppsala Sweden; ^2^ Växa Uppsala Sweden

**Keywords:** conformation, gaits, jumping, sport horses, young horse test

## Abstract

Swedish Warmblood horses (SWB) are bred for show jumping and/or dressage with young horse test scores as indicator traits. This study aimed to investigate possible candidate genes and regions of importance for evaluated and linearly scored young horse test traits. A single‐step genome‐wide association study (ssGWAS) was done using the BLUPF90 suite of programs for factors scores from factor analysis of traits assessed at young horse tests together with height at withers. The ssGWAS included 20,814 SWB with factors scores for four factors for evaluated traits. A total of 6436 of these horses also had factor scores for 13 factors for linearly scored traits. Genotypes from a 670K SNP array were available for 380 of the horses in this study. All genotyped horses had factor scores for evaluated traits, and 379 also had factors scores for linearly scored traits. Significant SNPs associated with three factors related to size were located on ECA3 within or nearby a well‐known region, including the genes *ligand dependent nuclear receptor corepressor like* (*LCORL*), *non‐SMC condensin I complex subunit G* (*NCAPG*), *DDB1 and CUL4 Associated Factor 16* (*DCAF16*), *and the Family with Sequence Similarity 184 Member B* (*FAM184B*). Significant SNPs were also detected for two factors for evaluated traits representing conformation and jumping, and four factors for linearly scored traits related to body length, neck conformation, walk and trot (hindleg position and activity), respectively. Among nearby genes, *calcium/calmodulin‐dependent protein kinase type 1D* (*CAMK1D*) for the factor for linearly scored traits related to neck conformation and *GLI Family Zinc Finger 2* (*GLI2*) for the factor for evaluated jumping traits, were most promising. For these, top associated SNPs were detected within the genes, and the known gene functions seems to be related to the phenotypes. In conclusion, ssGWAS is beneficial to detect plausible candidate genes/regions for desired traits in warmblood horses.

## 1 Introduction

Swedish Warmblood horses (SWB) are bred with the aim to produce noble, correct, durable and competitive horses at international level in show jumping and/or dressage (Swedish Warmblood Association 2021). Considerable genetic progress is seen for both show jumping and dressage performance (Bonow et al. 2023). However, there is limited knowledge about genomic regions or genes of importance for the young horse test traits that, together with competition performance, constitute the basis for the genetic evaluation (Viklund et al. 2011).

Identification of genomic regions, and potentially genes, with a significant impact on traits related to the breeding goal can increase our understanding of the biology and may influence future breeding strategies (Stock et al. 2016). The quality of phenotypic data is of crucial importance for genomic studies. During recent years, the more refined traits recorded using linear scoring protocols have been suggested to be well suited also for genomic analysis (Wobbe et al. 2021). A protocol for linear trait profiling was introduced in 2013 at young horse tests for 3‐year‐old SWB (Viklund and Eriksson 2018). In this scoring system, the horse is described on a linear scale from one biological extreme to another, for a range of separate traits. The linear profiling aims to describe, rather than evaluate traits, in contrast to the traditional evaluating system that score horses with regard to the breeding goal (Duensing, Stock, and Krieter 2014). Still, both types of traits are visually assessed by judges.

At present, 50 linearly scored traits are recorded at tests for 3‐year‐old SWB horses, in addition to height at withers and eight traditional evaluated traits. Several of them can be assumed to be highly correlated, and it is desirable to reduce the number of traits when conducting genomic studies of young horse test traits. Factor analysis (FA) is one of the methodologies that is used to shrink the number of correlated traits to a lower number of underlying factors that can be helpful to understanding the nature and biology of the traits. This method has been used for young horse test results for 3‐year‐old SWB horses in a study by Nazari‐Ghadikolaei et al. (2023), as well as in studies of other horse breeds and traits (Staiger, Albright, and Brooks 2016; Sigurðardóttir, Albertsdóttir, and Eriksson 2017).

Several genome‐wide association studies (GWAS) have been conducted in horses of warmblood sport horse type for conformation (Signer‐Hasler et al. 2012b; Frischknecht et al. 2016; Gmel et al. 2019; Reich et al. 2024), jumping (Schröder et al. 2012; Brard and Ricard 2015) and gaits (Legarra, Ricard, and Varona 2018; Ricard et al. 2020), including only genotyped horses. This has resulted in an increasing number of quantitative trait loci (QTLs), and gene(s), as reported in the Horse Quantitative Trait Locus Database (QTLdb 2023). However, far from all GWAS in horses have been successful in detecting significant associations and much work remains before a more complete understanding of the genetics behind traits related to show jumping and dressage performance is in place.

For horse populations where the recording of traits and pedigrees often has been extensive, whereas relatively few individuals are genotyped so far, the use of single‐step methods for GWAS can be valuable. Single‐step genomic best linear unbiased predictions (ssGBLUP) allows integration of genotype, phenotype and pedigree information simultaneously (Legarra et al. 2014). It can be implemented for conducting single‐step GWAS (ssGWAS) in two main steps, described by Wang et al. (2012): (1) prediction of genomic estimated breeding values (GEBV) and (2) obtain individual prediction error variances of marker effects to use in back solving genomic breeding values to SNP effects and to obtain *p* values. This method has an advantage of including both genotyped and non‐genotyped animals in the final model estimation. A few studies of traits in horses have used ssGWAS: Vosgerau et al. (2022) conducted ssGWAS to detect possible candidate loci or gene(s) associated with withers height in German Warmblood horses. Other examples of using ssGWAS include racing performance in Quarter horses (Pereira et al. 2018) and French trotters (Ricard and Duluard 2021), as well as gaits and performance in Brazilian Mangalarga Marchador horses (de Oliveira Bussiman et al. 2020).

The aim of this study was to conduct a ssGWAS of factors for evaluated and linearly scored traits in three‐year‐old SWB horses, to explore and detect possible candidate region(s) or gene(s) related to traits of importance for the breeding goal.

## 2 Material and Method

### 2.1 Animals and Phenotypes

In total, the data included records for 20,935 SWB horses assessed at young horse tests for 3‐year‐olds between 1999 and 2020. The data were provided together with a seven‐generation pedigree file by the Swedish Warmblood Association, via the Swedish University of Agricultural Sciences. All horses had evaluated trait records (scale from 1 to 10), and most (20,814) also had a measure of height at withers (cm). Among these horses, 6436 horses assessed between 2013 and 2020 also had linearly scored traits recorded (scale from 1 to 9). High density (670k) SNP data from Affymetrix Axiom Equine Genotyping Array, previously used by Ablondi, Viklund, et al. (2019), was available for 380 of the horses (182 males and 198 females) in the present study. The genotyped horses were all assessed at young horse tests for 3‐year‐olds during the years 2013 and 2014 and had complete information about height at withers and all evaluated traits. In addition, 379 of the genotyped horses had linearly scored trait records. Each horse was only assessed once, so there were no repeated scores. The 380 genotyped horses descended from 145 sires with 1–11 offspring each and 372 mares with 1–2 offspring each. As described by Ablondi, Viklund, et al. (2019), the horses were selected for genotyping on the basis of their young horse test results for evaluating traits. Selected horses had either high scores for jumping but lower scores for gaits (*N* = 48), high scores for gaits but lower scores for jumping (*N* = 48), high scores for both jumping and gaits (*N* = 143), or low scores for both jumping and gaits (*N* = 141). Efforts were made to match half‐sibs across different trait profiles in this data set.

### 2.2 Factor Analysis

Factor analyses were done using the Psych package (Revelle 2022) in R (R Core Team 2019) for complete records for evaluated traits together with height at withers, and for linearly scored traits together with height at withers, as described in detail by Nazari‐Ghadikolaei et al. (2023). The correlation matrix used in the factor analysis was approximated by the product of a factor matrix and its transpose plus a diagonal matrix of uniqueness (proportion of variance not explained by the common factor) (Revelle 2022). Factors were extracted using maximum likelihood and subsequently rotated by the ‘varimax’ rotation to produce more easily interpreted factors that represented a smaller number of variables (Abdi 2003). The optimum number of factors was determined as the number of factors with Tucker Lewis Index larger than 0.9 and root mean square of residuals (RMSR index) value of close to zero (Tucker and Lewis 1973; Steiger 1990), as well as eigenvalues above 1.

To calculate factor scores, we used the factor loadings, the inverse of the data covariance matrix and the trait data after imputation and applied the regression method (Thurstone 1935). In contrast to the study by Nazari‐Ghadikolaei et al. (2023), the first year of linear scoring (2013) was included in this study, and the traits related to conformation of pasterns (for which information was lacking in 2013) were removed, but this had a very small effect on the formation of factors, other than removing a specific factor for pasterns. To be able to predict factor scores in spite of a few missing linearly scored trait observations, missing values were imputed on the basis of correlated traits using the Mice package (Van Buuren and Groothuis‐Oudshoorn 2011) in R as outlined in Nazari‐Ghadikolaei et al. (2023). For all factors for which results are presented in this manuscript, the traits with significant loadings (≥ |0.3|) were available for on average 99% of the genotyped horses (ranging from 94% to 100% for the different linearly scored traits).

Four factors for evaluated traits and 13 factors for linearly scored traits were used for the ssGWAS. More details about the factors are given when the results are presented and discussed later in this article and in Nazari‐Ghadikolaei et al. (2023).

### 2.3 Genotype Quality Control

Genotype quality control (QC) was performed in PLINK v.1.9 (Purcell et al. 2007), removing animals and SNPs with less than 90% genotyping call rates, SNPs with minor allele frequency (MAF) less than 0.05, and SNPs with *p* value for Hardy–Weinberg Equilibrium (HWE) test less than 1e‐6 from further analyses. In total, 30,712 SNPs were removed because of low call rate, 18,933 were removed on the basis of HWE, and 197,375 SNPs were removed on the basis of low MAF. Finally, 380 animals with 389,997 SNPs remained for the ssGWA study.

### 2.4 Single‐Step Genome‐Wide Association Study

Prediction of genomic breeding values (GEBVs) was done as the first step in the ssGWAS. The estimation of genetic parameters (Table S1) that was used for this purpose is described in Nazari‐Ghadikolaei et al. (2023). The GEBVs were predicted using BLUPF90 (Misztal et al. 2002) with a single‐trait animal model, including the same fixed effects (sex and event) as was used for the estimation of genetic parameters. In the single‐step prediction, the inverse of a hybrid relationship matrix, **H** ^−1^, was used instead of the inverse numerator relationship matrix **A** ^−1^ as follows (Aguilar et al. 2010):

$$H^{-1}=A^{-1}+\left[\begin{array}{cc} 0 & 0\\ 0 & \;G^{-1}-A_{22}^{-1} \end{array}\right]$$

where **A** is the pedigree‐based numerator relationship matrix for all animals, **G** is a genomic relationship matrix, and **A**_**22**_ is a numerator relationship matrix for genotyped animals. The default weight of 0.95 on genomic information was used. After prediction of GEBVs, POSTGSF90 was used for estimating SNP effects (Wang et al. 2012; Aguilar et al. 2014), % variance explained by each SNP with OPTION windows_variance with a window size of 1 SNP, and *p* values using OPTION *p* value according to (Aguilar et al. 2019).

Quantile–quantile (QQ) and Manhattan plots were created using CMplot package in R (Yin 2022), The QQ‐plots were used to interpret the inflation factor (lambda value) because of possible cryptic relationship among animals and to detect significant SNPs. Estimation of lambda value was done using the estlambda() function in the GenABEL package in R (Aulchenko et al. 2007; R Core Team 2022). Significant SNPs were identified on the basis of Bonferroni significant cut‐off calculated as (0.05/389,997 = 1.28E‐07), and for all traits with such associated SNPs in this study, the lambda value was ≤ 1.0. For SNPs that failed to pass the Bonferroni cut‐off, a *p* value adjustment for a lambda value > 1.0 was done, and a false discovery rate (FDR) was estimated using Benjamini–Hochberg (BH) method in GenABEL package (Benjamini and Hochberg 1995; Aulchenko et al. 2007). SNPs with FDR values ≤ 0.3 were considered as a significant. This FDR threshold is inclusive, but was found to give similar *p* value levels for most factors to what has commonly been used for suggestive significance in GWA studies in horses for example by Gmel et al. (2019) and Frischknecht et al. (2016). Moreover, linkage disequilibrium between significant SNPs on the same chromosome was calculated using the Gaston package in R (Perdry and Dandine‐Roulland 2022), with the *r* ^2^ method.

### 2.5 Functional Annotation

Putative candidate QTL(s) or gene(s) were investigated on the basis of ±500‐kb windows around the detected significant SNPs, using EquCab 3.0 (GCF_002863925.1) assembly (Kalbfleisch et al. 2018) in Genome Data Viewer on National Centre for Biotechnology Information (NCBI) and BioMart on Ensemble (Sayers et al. 2022; Martin et al. 2023).

### 2.6 Least Squares Means

To illustrate the association of the top SNPs detected to be significant with factors in this study, least squares means were estimated using the mixed procedure in the SAS software (SAS Institute Inc. 2015). The effect of event (*N* = 50) and sex (male or female) and one top SNP at the time was included in the model for each trait. Three event classes with fewer than three genotyped horses each were merged with classes of events held at the same place but in a different year. This was only done for the estimation of least squares means and not for the ssGWAS, because the number of observations per event was reduced when including only genotyped horses.

## 3 Results

Significant associations based on either Bonferroni correction or FDR after correction for the lambda value were found for three of the four factors for evaluated traits and for six of the 13 studied factors for linearly scored traits, and those are presented below. Estimated least squares means are shown in Table S3 and Figure S2, and illustrate the size of the impact from genotypes for the detected top SNPs on the phenotypes in this data. In most cases, the least squares mean estimates points at substantial differences in relation to size of the standard deviations (Table S1) for the factor traits. However, likely due to low genotype frequency in some cases, not all genotypes had significantly different effects.

### 3.1 Factors Related to Body Height and Size

**FIGURE 1:**
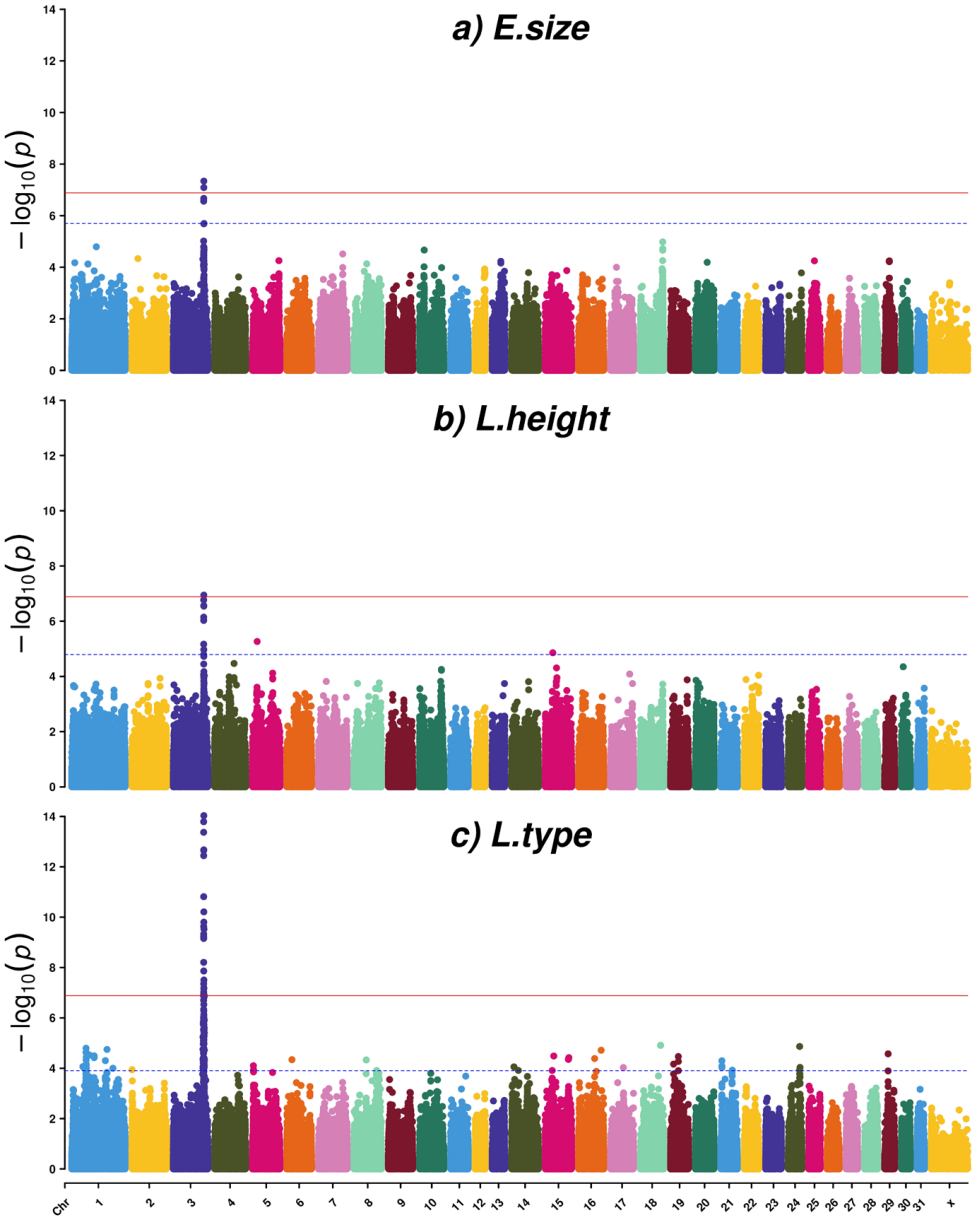
Manhattan plots for three factors for evaluated (E) and linearly scored (L) traits related to body height and size in SWB horses: (a) E.size, (b) E.height and (c) L.type. The red line indicates the Bonferroni corrected significance threshold (0.05/389,997 = 1.28E‐07), and the blue dotted line indicates the FDR (≤ 0.3) significance threshold. [Colour figure can be viewed at wileyonlinelibrary.com]

**FIGURE 2:**
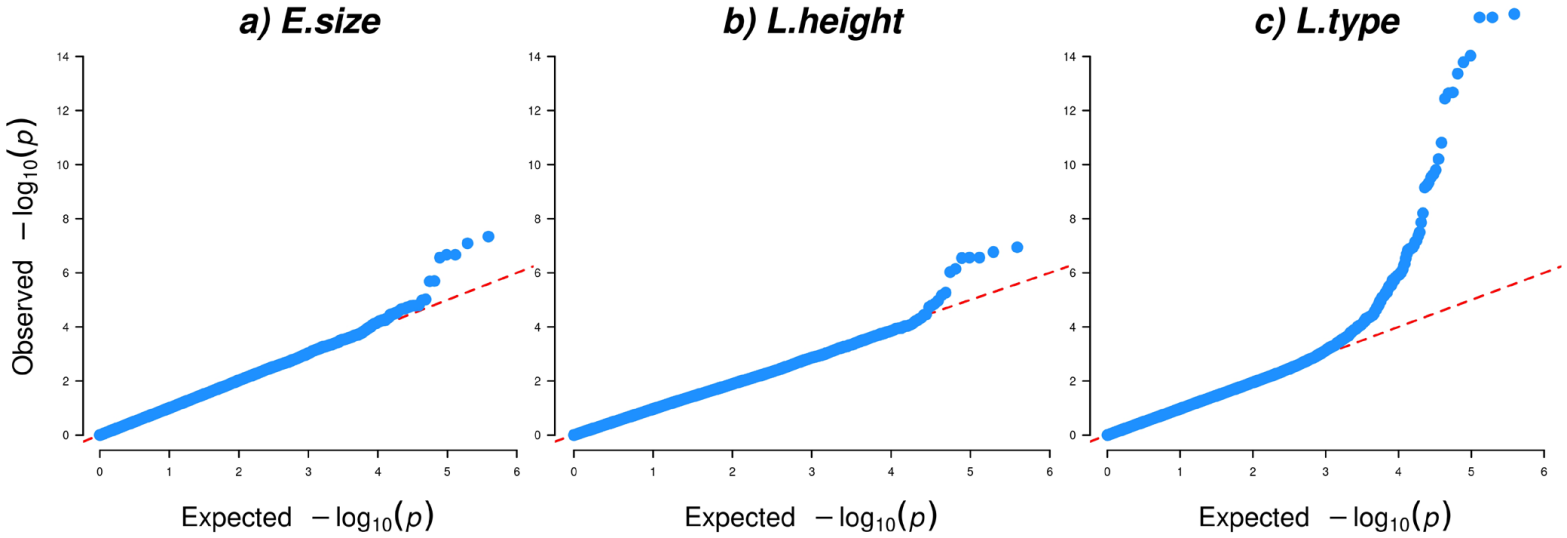
Quantile–quantile (QQ) plots for three factors for evaluated (E) and linearly scored (L) traits related to body height and size in SWB horses: (a) E.size, (b) E.height and (c) L.type. [Colour figure can be viewed at wileyonlinelibrary.com]

For the factor E.size, where height at withers and the evaluated traits type and head–neck‐body were the most important traits, two SNPs (AXE‐104376260 and AXE‐103658351) surpassed the Bonferroni cut‐off significance level (Table 1, Table S2, Figure 1a [Manhattan plot] and Figure 2a [QQ‐plot]). Both SNPs were located on ECA3, in genomic position 107,641,233 and 107,643,810 bp, respectively. The LD between these two SNPs was estimated as 0.72 (Figure S1a). The top SNP for E.size (AXE‐104376260) was located within 3′UTR of the *DDB1 and CUL4 Associated Factor 16* (*DCAF16*) gene and overlapping 5′UTR of the *Family with Sequence Similarity 184 Member B* (*FAM184B*) gene.

**TABLE 1:** Associated SNP(s) with FDR ≤ 0.3 for factors in 380 SWB horses and nearest annotated protein coding genes within ±500 kb from the SNP(s).

Factor	*λ* ^a^	ECA^b^	Top SNP				All significant SNP(s) in the region		
			Marker name	Pos. (bp)	FDR^c^	*p* ^d^	Position range (bp)	N SNPs^e^	Protein coding gene(s)^f^
E.size	1.00	3	AX‐104376260	107,641,233	0.02	4.56E‐08^g^	107,641,233–107,643,810	2	*LCORL, NCAPG, **DCAF16, FAM184B**, MED28, LAP3, CLRN2, QDPR*
L.height	0.94	3	AX‐104376260	107,641,233	0.01	1.13E‐07^g^	107,641,233	1	*LCORL, NCAPG, **DCAF16, FAM184B**, MED28, LAP3, CLRN2, QDPR*
L.type	0.99	3	AX‐103036686	107,115,309	3.4E‐11	2.67E‐16^g^	106,606,527–108,232,943	26	***LCORL**, NCAPG, **DCAF16, FAM184B**, MED28, LAP3, **CLRN2**, QDPR, LDB2*
L.length	1.10	15	AX‐104176662	5,021,795	0.13	3.26E‐07	5,021,795	1	*TGFBRAP1, GPR45, MRPS9, POU3F3*
E.conf	1.14	26	AX‐105004994	30,188,501	0.29	7.50E‐07	30,188,501	1	*SOD1, SCAF4, HUNK, ENSECAG00000037230, MIS18A, MRAP, URB1, EVA1C, CFAP298‐TCP10L, SYNJ1*
L.neck	1.17	29	AX‐103450830	23,384,193	0.19	4.81E‐07	23,384,193–23,400,348	2	*CCDC3, **CAMK1D**, CDC123, NUDT5, SEC61A2, DHTKD1, UPF2, PROSER2, ECHDC3, USP6NL*
		14	AX‐103936375	17,446,349	0.28	1.47E‐06	17,446,349	1	*GABRA1, GABRA6, GABRB2*
E.jump	1.79	18	AX‐105015252	9,956,043	0.14	3.62E‐07	9,956,043	1	*CLASP1, TFCP2L1, ENSECAG00000051759, **GLI2***
L.walk	1.18	17	AX‐104203221; AX‐105012856	75,955,220; 75,955,247	0.29	2.22E‐06	75,955,220–75,955,247	2	*NALF1, LIG4, ABHD13, TNFSF13B, MYO16*
		22	AX‐104667493	28,543,491	0.29	1.86E‐06	28,543,491	1	*RAB5IF, SLA2, NDRG3, SOGA1, TLDC2, SAMHD1, RBL1, MROH8, RPN2, GHRH, MANBAL, SRC, SOGA1, TLDC2, SAMHD1, RBL1, MROH8, RPN2, GHRH, MANBAL, SRC, ENECAG00000052590, BLCAP, NNAT, CTNNBL1*
L.trot.hind	1.35	15	AX‐103369307	3,871,175	0.23	5.83E‐07	3,871,175	1	*UXS1, ECRG4, NCK2, ENSECAG00000051693*

^a^ Lambda value before correction.

^b^ Equine chromosome number.

^c^ False discovery rate.

^d^ Adjusted for lambda value when *λ* > 1.

^e^ Number of significant SNPs in the region.

^f^ Genes in which any significant SNPs are located are marked in bold.

^g^ Surpassed the Bonferroni significant threshold.

The same SNP AXE‐104376260 also surpassed the Bonferroni significance cut‐off for the factor L.height, dominated by height at withers and the linearly scored traits withers (high to low), and body proportions (long legged to short legged) (Table 1, Table S2 and Figures 1b and 2b). This SNP was in LD with two other close/neighbour SNPs: AXE‐103658351 and AXE‐103175342 with LD‐values of 0.72 and 0.76, respectively, and those SNPs were also significantly associated with L.type (Figure S1a).

For the factor L.type for which height at withers and the linearly scored trait type (refined to heavy) loaded most strongly, 26 SNPs located in the genomic region ECA3:g.106,606,527—108,232,943, including both SNPs mentioned above detected for E.size and L.height, surpassed the Bonferroni significance cut‐off (Table 1, Table S2 and Figures 1c and 2c). The LD between all 26 SNPs ranged from 0.04 to 0.99 (Figure S1a). The top SNP AX‐103036686 for L.type was located at ECA3:g.107,115,309 bp, equivalent to 318‐kb upstream of the *ligand dependent nuclear receptor corepressor like* (*LCORL*) gene. Three other significantly associated SNPs for L.type (AX‐102966003, AX‐103096129 and AX‐103175342) are located in introns within the *LCORL* gene (at positions ECA3:g.107,456,470, ECA3:g.107,496,015 and ECA3:g.107,528,272 bp). Besides the SNP AX‐104376260 within *DCAF16* (*3′UTR*) *and FAM184B* (*5′UTR*), three other significant SNPs were located within introns of the *FAM184B* gene at ECA3:g.107,643,810, ECA3:g.107,685,587 and ECA3:g.107,702,189 bp. Also, the significant SNP AX‐104783182 at ECA3:g.107,852,187 bp was located in the 3′UTR of the gene *clarin 2* (*CLRN2*) (Table S2).

Other genes within ±500 kb to the significant SNPs for the three factors related to body size, especially height, were the *non‐SMC condensin I complex subunit G* (*NCAPG*), *mediator complex subunit 28* (*MED28*), *leucine aminopeptidase 3* (*LAP3*) *and quinoid dihydropteridine reductase* (*QDPR*) (Table 1).

### 3.2 Factor Related to Body Length

**FIGURE 3:**
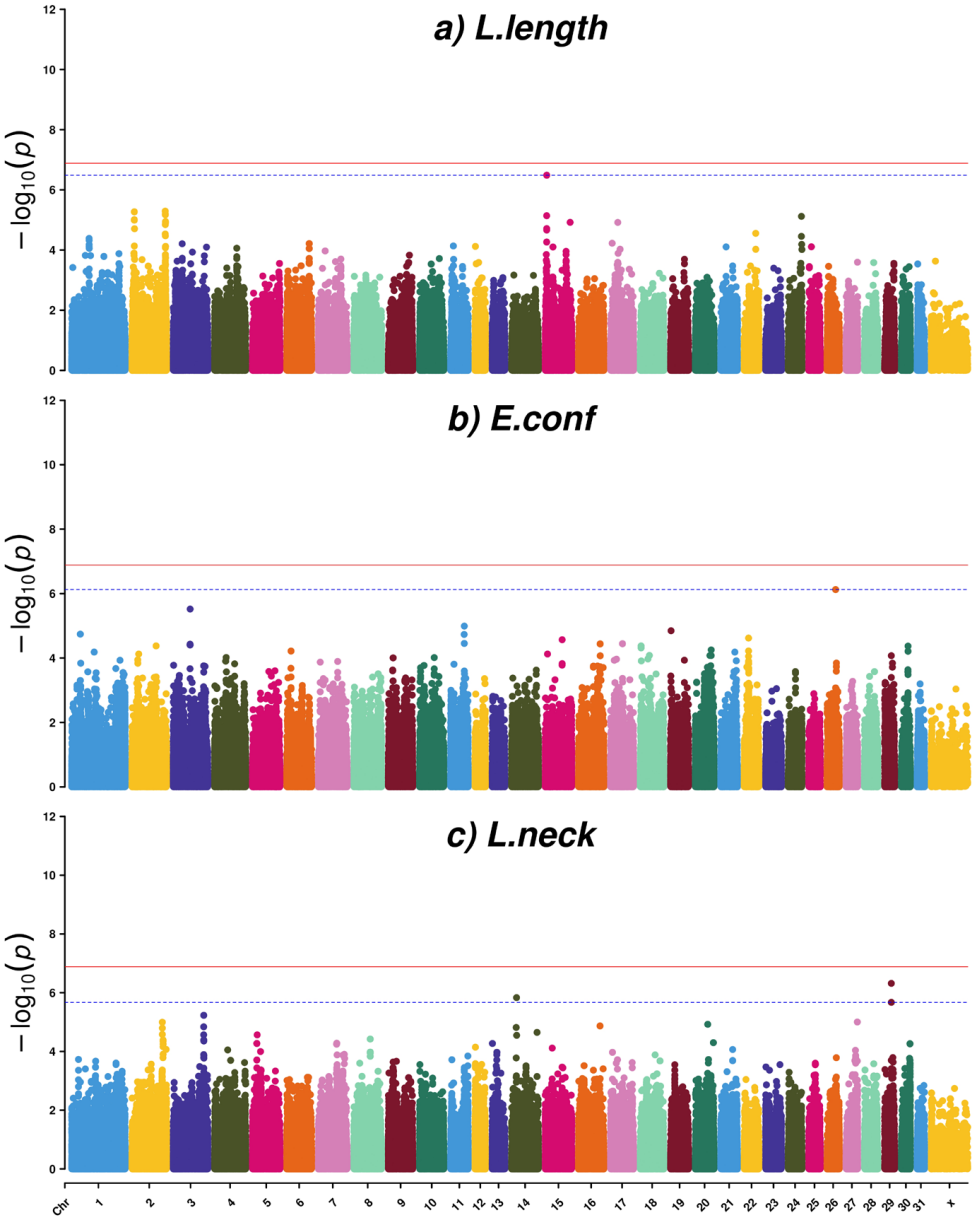
Manhattan plots for factors for (a) linearly scored body length traits (L.length), (b) evaluated conformation traits (E.conf) and (c) linearly scored neck conformation traits (L.neck), in SWB horses. The red line indicates the Bonferroni corrected threshold (0.05/389,997 = 1.28E‐07), and the blue dotted line indicates the FDR (≤ 0.3) significance threshold. [Colour figure can be viewed at wileyonlinelibrary.com]

**FIGURE 4:**
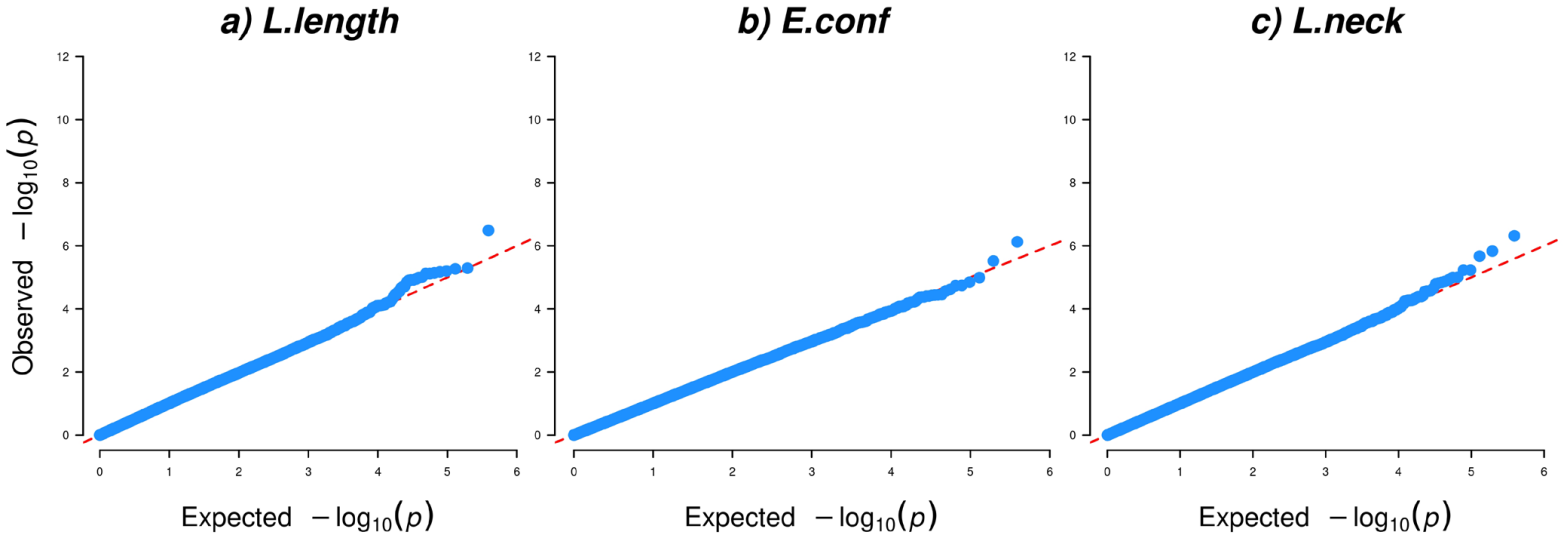
Quantile–quantile (QQ) plots for factors for (a) linearly scored body length traits (L.length), (b) evaluated conformation traits (E.conf) and (c) linearly scored neck conformation traits (L.neck), in SWB horses. [Colour figure can be viewed at wileyonlinelibrary.com]

For the factor L.length, comprising the linearly scored traits body length (long to short), loins (long to short) and length of neck (long to short), the most significant SNP (AXE‐104176662) was located on ECA15:g.5,021,795 bp. This SNP surpassed the FDR significance level with a FDR value of 0.13 (Table 1, Table S2 and Figures 3a and 4a) and was upstream of *transforming growth factor‐beta receptor associated protein 1* (*TGFBRAP1*), *G protein‐coupled receptor 45* (*GPR45*), *mitochondrial ribosomal protein S9* (*MRPS9*) and *POU class 3 homeobox 3* (*POU3F3*) (Table 1).

### 3.3 Factors Related to Other Conformation Traits

For the factor E.conf, dominated by the evaluated traits type and head–neck‐body, one SNP (AX‐105004994) located on ECA26:g.30,188,501 bp passed the FDR threshold with a FDR value of 0.29 (Table 1, Table S2 and Figures 3b and 4b). The top SNP for this factor was located intergenic, in a region comprising the genes *superoxide dismutase 1* (*SOD1*), *SR‐related CTD associated factor 4* (*SCAF4*), *hormonally up‐regulated Neu‐associated kinase* (*HUNK9*), *ENSECAG00000037230*, *MIS18 kinetochore protein A* (*MIS18A*), *melanocortin 2 receptor accessory protein* (*MRAP*), *URB1 ribosome biosis homologue* (*URB1*), *eva‐1 homologue C* (*EVA1C*), *CFAP298‐TCP10L readthrough* (*CFAP298‐TCP10L*) and *synaptojanin 1* (*SYNJ1*) (Table 1).

Three SNPs passed the FDR significance threshold for the factor L.neck dominated by the linearly scored traits position of neck (vertical to horizontal), shape of neck (arched to straight) and position of shoulder (sloping to straight). Two of the SNPs (AX‐103450830 and AX‐104958036) were located on ECA29: g.23,384,193 and ECA29: g.23,400,348 bp, and had FDR values of 0.19 and 0.28, respectively (Table 1, Table S2 and Figures 3c and 4c). The LD value between these two SNPs was 0.89 (Figure S1b). These two SNPs were located within the *calcium/calmodulin‐dependent protein kinase ID* (*CAMK1D*) *gene*. Besides that, nearby genes included *coiled‐coil domain containing 3* (*CCDC3*), *cell division cycle 123* (*CDC123*), *nudix hydrolase 5* (*NUDT5*), *SEC61 translocon subunit alpha 2* (*SEC61A2*), *dehydrogenase E1 and transketolase domain containing 1* (*DHTKD1*), *UPF2 regulator of nonsense mediated mRNA decay* (*UPF2*), *proline and serine rich 2* (*PROSER2*) and *enoyl‐CoA hydratase domain containing 3* (*ECHDC3*) (Table 1).

The third SNP (AX‐103936375) that passed the FDR threshold for L.neck was located in an intergenic region on ECA14:g.17,446,349 bp (Table 1 and Figures 3c and 4c) and had an FDR value of 0.28 (Table S2). The nearest genes to this SNP were *gamma‐aminobutyric acid type A receptor subunit alpha1* (*GABRA1*), *gamma‐aminobutyric acid type A receptor subunit alpha6* (*GABRA6*) and *gamma‐aminobutyric acid type A receptor subunit beta2* (*GABRB2*), *and* the detected SNP was located upstream of the *GABRB2* gene (Table 1).

### 3.4 Factor for Jumping

**FIGURE 5:**
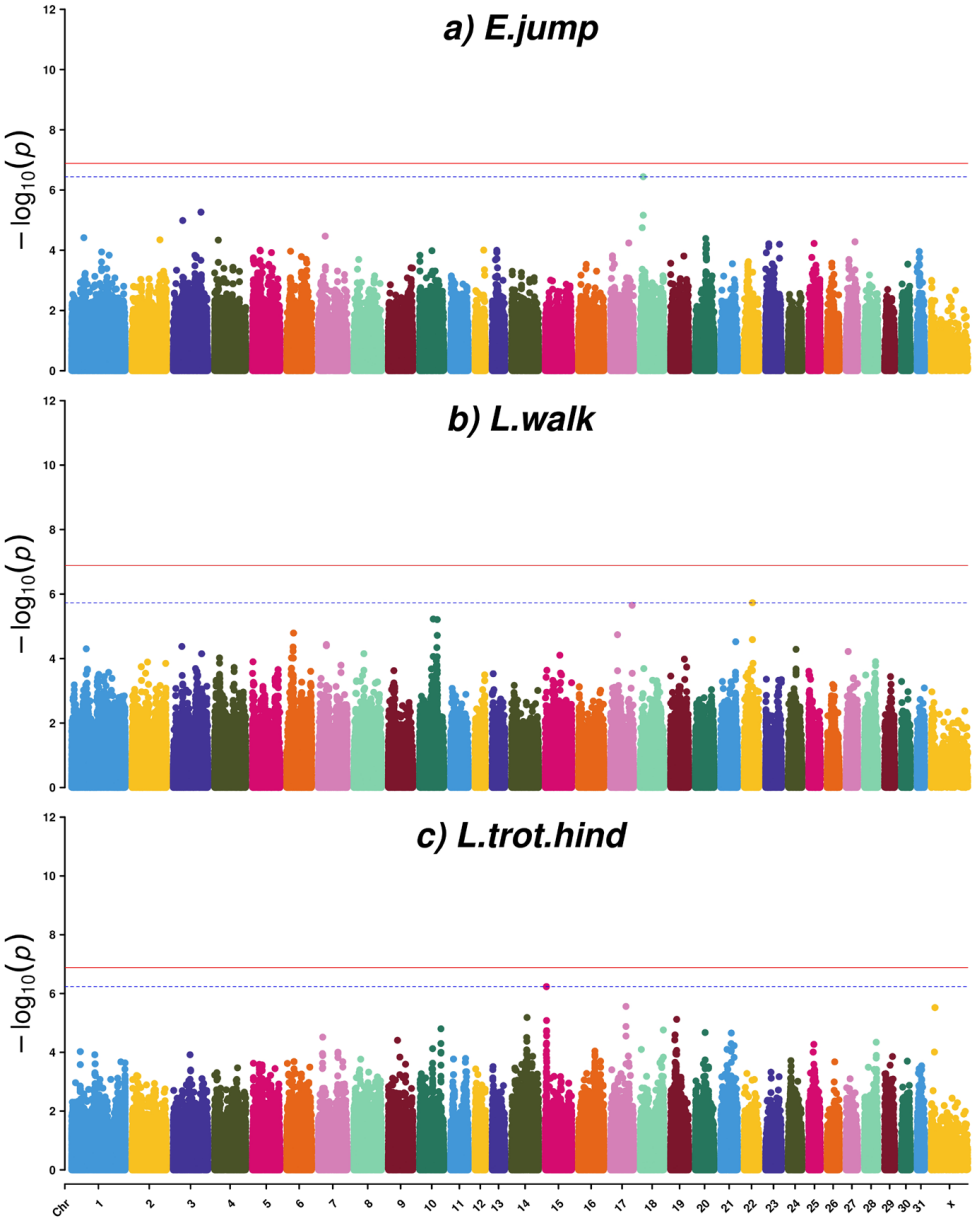
Manhattan plots for factors for (a) evaluated jumping traits (E.jump), (b) linearly scored walk traits (L.walk) and (c) linearly scored trot traits with focus on the hind legs (L.trot.hind), in SWB horses. The red line indicates the Bonferroni corrected threshold (0.05/389,997 = 1.28E‐07), and the blue dotted line indicates the FDR (≤ 0.3) significance threshold. [Colour figure can be viewed at wileyonlinelibrary.com]

**FIGURE 6:**
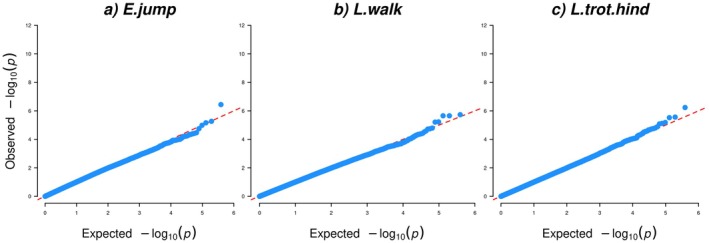
Quantile–quantile (QQ) plots for factors for (a) evaluated jumping traits (E.jump), (b) linearly scored walk traits (L.walk) and (c) linearly scored trot traits with focus on the hind legs (L.trot.hind), in SWB horses. [Colour figure can be viewed at wileyonlinelibrary.com]

For the factor E.jump, collecting the two evaluated traits free‐jumping technique and ability, and free‐jumping temperament and general impression, one SNP (AX‐105015252) in position ECA18:g.9,956,043 bp passed the FDR significance level, with a value of 0.14 (Table 1, Table S2 and Figures 5a and 6a). This SNP had a LD of 0.76 with its nearby SNP (AX‐104534955) (Figure S1c). These SNPs were located within the *GLI family zinc finger 2* (*GL2*) gene. Other genes close to the top SNP included *cytoplasmic linker associated protein 1* (*CLASP1*) and *transcription factor CP2 like 1* (*TFCP2L1*) *and ENSECAG00000051759* (Table 1).

### 3.5 Factors for Walk and Trot

Three SNPs surpassed the FDR significance threshold with the FDR value of 0.29 for the factor L.walk that was dominated by the linearly scored traits walk stride length (long to short), walk cadence (even to uneven) and walk suppleness (supple to stiff) (Table S2). Two of these SNPs (AX‐104203221 and AX‐105012856) were completely in LD with each other and located intergenic on ECA17: g.75,955,220 and ECA17: g.75,955,247 bp, respectively (Table 1, Figures 5b and 6b, and Figure S1d). The closest genes in the ±500 kb window included *DNA ligase 4* (*LIG4*), *abhydrolase domain containing 13* (*ABHD13*), *TNF superfamily member 13b* (*TNFSF13B*) and *myosin XVI* (*MYO16*) (Table 1).

The third SNP that passed the FDR threshold for L.walk (AX‐104667493) was located intergenic on ECA22:g.28,543,491 bp (Table 1 and Figures 5b and 6b). This chromosome region is quite gene dense and involves the genes *RAB5 interacting factor* (*RAB5IF*), *Src like adaptor 2* (*SLA2*), *NDRG family member 3* (*NDRG3*), *suppressor of glucose autophagy* (*SOGA1*), *TBC/LysM‐associated domain containing 2* (*TLDC2*), *SAM and HD domain containing deoxynucleoside triphosphate triphosphohydrolase 1* (*SAMHD1*), *RB transcriptional corepressor like 1* (*RBL1*), *maestro heat like repeat family member 8* (*MROH8*), *ribophorin II* (*RPN2*), *growth hormone releasing hormone* (*GHRH*), *mannosidase beta like* (*MANBAL*), *SRC proto‐onco non‐receptor tyrosine kinase* (*SRC*), *ENECAG00000052590*, *BLCAP apoptosis inducing factor* (*BLCAP*), *neuronatin* (*NNAT*) and *catenin beta like 1* (*CTNNBL1*) were the closest genes to this SNP (Table 1).

Finally, for the factor L.trot.hind on which the linearly scored traits trot hind leg activity (active to inactive), trot hind leg position (under the body to behind the body) and trot elasticity (elastic to inelastic) loaded most strongly. One FDR significant SNP (AX‐103369307) located intergenic on ECA15:g.3,871,175 bp with the FDR value of 0.23 was found (Table 1, Table S2 and Figures 5c and 6c). The genes *UDP‐glucuronate decarboxylase 1* (*UXS1*), *ECRG4 augurin* (*ECRG4*) and *NCK adaptor protein 2* (*NCK2*) and *ENSECAG00000051693* were found within ±500‐kb window (Table 1).

## 4 Discussion

In this study, single‐step GWAS was used to identify plausible regions by estimating SNP effects and *p* values, including also non‐genotyped horses. The analysis was based on factors instead of all individual evaluated and linearly scored traits. This was done to reduce the number of traits to a manageable number of latent traits with a meaningful interpretation (Nazari‐Ghadikolaei et al. 2023). It has been demonstrated that estimated marker effect divided by its standard error obtained from GBLUP and standard single‐marker GWAS methods are equivalent (Gualdrón Duarte et al. 2014; Aguilar et al. 2019; Legarra, Ricard, and Varona 2018). The single‐step approach in which genomic breeding values were predicted and then back solved to SNP effects and *p* values, enabled adjustments of the phenotypes for effects in the model as well as correction for genetic structure in the population. It also made it possible to utilise phenotypic information from a large number of horses that were related to the relatively few genotyped individuals included in this study, which makes the use of single‐step methodology attractive for this type of data.

It should be noted that all the studied traits are complex traits, based on subjective measures and likely influenced by many genes and environmental influence, and that some missing factor scores were also obtained by imputation. This, in combination with the relatively small number of genotyped individuals, only allows for detection of genomic regions with large influence on the trait. In the end, the factors for which we found significant associations were not depending heavily on traits for which many observations had to be imputed, and thus, the effect of imputation for the presented results can be assumed to be minor.

The Bonferroni method is a simple and common correction for multiple testing, but it does not consider that some SNPs are in high LD with each other and thus not independent. Also, the method to minimise the family‐wise error rate has been suggested to be too strict in many multiple test settings (Noble 2009). In contrast, to control the FDR has been proposed to be a reliable choice in the context of multiple testing, that reduces the risk of unnecessarily removing potentially important results (Glickman, Rao, and Schultz 2014). For this reason, we chose to use the less conservative FDR threshold in this study, in addition to the well‐known Bonferroni corrected significance level. This FDR threshold gave similar suggestive associations as applying *p* value levels commonly used for suggestive significance in GWA studies in horses for example by Gmel et al. (2019) and Frischknecht et al. (2016).

The percentage of variance explained according to the results from the ssGWAS (Table S2) was in general considerably smaller for individual top SNPs than what has been indicated from previous studies, for example, for SNPs in the vicinity of the *LCORL* gene for height at withers by Vosgerau et al. (2022) who also used a single‐step approach but another software. The difference is due to the different methods used and it should be noted that many SNPs in the region together explain more of the variation, and those were not considered in the calculation of the percentage of variance explained. To illustrate that the detected top SNPs did in fact seem to have a substantial influence on the studied factors, we included also estimations of least squares means where only genotypes for the top SNP were included for each trait, and then, a larger effect was as expected observed.

### 4.1 Factors Related to Body Height and Size

SNPs in the same region on ECA3 were detected for the three factors related to body size, primarily height at withers: E.size, L.height and L.type. This region contains the *LCORL* and *NCAPG* genes, reported to influence height and size variation in horses and other species (Pryce et al. 2011; Signer‐Hasler et al. 2012a; Tetens et al. 2013; Staiger et al. 2016; Reich et al. 2024). In horses, a lower expression level of *LCORL* may be associated with larger and heavier horses (Metzger et al. 2013). Moreover, a well‐documented QTL close to *LCORL* gene has been identified for withers height using ssGWAS in German Warmblood horses (Vosgerau et al. 2022). A recent comprehensive study of German Warmblood horses by Reich et al. (2024) also reported a possible, albeit less likely, effect of the gene *DCAF16* in addition to *LCORL* and *NCAPG* for body size.

The *LCORL* and *NCAPG* genes in addition to *FAM184B* gene have also been associated with conformation and locomotive traits in Spanish purebred horses (Sevane et al. 2017). Also, a study of Simmental cattle showed possible associations between *LCORL, NCAPG* and *FAM184B* genes with bone weight in this breed (Xia et al. 2017). In another study, the *DCAF16*‐*NCAPG* region was identified as potential candidate region responsible for average daily weight gain in Simmental beef cattle (Zhang et al. 2016).

### 4.2 Factor Related to Body Length

Association mapping for the factor L.length, based on different traits describing length of the horse, could identify three genes on ECA15 within ±500 kb from the FDR significantly associated SNP. One of the genes was *TGFBRAP1*, which encodes a protein that binds to *transforming growth factor‐beta* (*TGF‐beta*) receptors, and plays a key role in *TGF‐beta* signalling pathway, which was one of the overrepresented gene ontology terms in SWB non‐show jumping horses (Ablondi, Eriksson, et al. 2019). The TGF‐beta superfamily plays important roles as morphogens during embryogenesis and is involved in tissue differentiation and in the establishment of body‐axes (Messler et al. 2011), which makes it a possible candidate for a trait related to the length of the longitudinal axis. *TGF‐beta* was previously found to play a role for muscle development and feed efficiency in beef cattle (Alexandre et al. 2019; Chen et al. 2021). Also, the *POU3F3* gene, located nearby, is important for embryo development (Safran et al. 2021).

### 4.3 Factors Related to Other Conformation Traits

For the E.conf factor dominated by the evaluated traits type and head–neck‐body, association mapping could detect nine genes on chromosome 26 within ±500 kb from the FDR significantly associated SNP. Among detected genes, the *EVA1C* gene is related to X‐Linked Intellectual Disability‐Short Stature‐Overweight Syndrome. Some of the symptoms of the syndrome include short stature, elevated body mass index, a pattern of truncal obesity (in older males), and variable neurologic features (Rappaport et al. 2013). As *EVA1C* has some association with short stature and body mass index, and E.conf is composed of type and head–neck‐body of the horse, it may be interesting to investigate whether *EVA1C* could be of importance for conformation in SWB horses.

For the L.neck factor based on traits describing the shape and position of neck and shoulders, nine genes were identified within the ±500 kb window on ECA29. The two top SNPs were located within the *CAMK1D* gene, and a member of the calcium/calmodulin‐dependent protein kinase 1 family, a subfamily of the serine/threonine kinases. The encoded protein is a component of the calcium‐regulated calmodulin‐dependent protein kinase cascade (Safran et al. 2021). *CAMK1D* has been suggested to play a role for liver gluconeogenesis, fat mass deposition, obesity and reduced insulin sensitivity (Rausch et al. 2018; Fromont et al. 2020). If fat deposition around the neck contributes to a more rounded/arched neck shape in SWB horses, then the *CAMK1D* could be a candidate gene for this trait. Enlarged fat depositions at the crest of the neck appear to be associated with insulin resistance and risk of laminitis in horses (Geor 2008; Carter et al. 2009).

The nearby *UPF2* and *ECHDC3* genes were suggested to be associated with pin width in Chinese Holstein cattle (Lu et al. 2021), and the *ECHDC3* gene plays a role for fatty acid biosynthesis and insulin sensitivity (Safran et al. 2021). Thus, this ECA29 region could be important for energy metabolism in the horse.

A potential genetic connection between the shape of the neck of horses and fat deposition and energy metabolism has been suggested previously. Gmel, Brem, and Neuditschko (2023) found in Lipizzan horses that a SNP within the gene, *Membrane Associated Guanylate Kinase, WW And PDZ Domain Containing 1* (*MAGI1*) on ECA16, was significantly associated with the shape and width of the neck. The *MAGI1* gene has also been suggested to be associated with insulin resistance and glucose response in humans (Palmer et al. 2010; Norris and Rich 2012; Ellis et al. 2015).

Also, the skeletal framework of the neck can be assumed to influence its conformation. In the present study, among the genes nearby the associated SNP on chromosome 14, the genes *GABRA1*, *GABRB2* and, especially, *GABRA6* have previously been suggested to be associated with osteochondrosis in Standardbreds (McCoy et al. 2016), and Hanoverian warmblood horses (*GABRA6*) (Naccache, Metzger, and Distl 2018). Osteochondral fragmentation of the cervical articular process joints in the neck of horses has been reported (Tucker et al. 2022). These genes are otherwise known for their importance in the central nervous system (Safran et al. 2021).

### 4.4 Factor for Jumping

The SNP located within the *GLI2* gene on ECA18 passed FDR significance threshold for the factor E.jump, incorporating jumping ability, technique, attitude and general impression. This gene codes for the transcription factor Zinc finger protein GLI2, that is mediating the Sonic hedgehog (Shh) signalling, and is thought to play a role during embryogenesis (Safran et al. 2021).

Loss of function in the *GLI2* gene has been shown to cause microphthalmia and decreased head width in homozygous mice (Heyne et al. 2016). The *GLI*‐mediated *CyklinD2* is important for the development of the binocular circuit, when retinal ganglion cells (RGC) from the retina project axons to the brain (Slavi et al. 2023). The same study by Slavi et al. (2023) found that the depth perception was compromised in CyklinD2‐deficient mice. It would be interesting to study further whether the *GLI2* gene could be of importance for distance estimation in show jumping horses.

The window of ±500 kb from the significant SNP also included the gene *CLASP1*, for which mutations have been related to disorders, such as epiphyseal dysplasia that affect cartilage and bone development, especially in the long bones in the limbs, short stature, microcephaly and congenital nystagmus (Safran et al. 2021).

### 4.5 Factors for Walk and Trot

For L.walk, based on traits describing different aspects of the gait walk, there were five genes in the studied window around the top SNPs on ECA17 and ECA22, and 25 genes in the ±500‐kb window on ECA22. Among the detected genes on ECA17, *ABHD13* is related to the rare Williams syndrome in humans that cause growth delay, short stature, mental deficiency and some facial features developing with age (Safran et al. 2021). Another nearby gene *TNFSF13B* encodes the cytokine protein belonging to the tumour necrosis factor (TNF) ligand family, and has been associated with body weight gain in beef steers (Lindholm‐Perry et al. 2017). Also, the *MYO16* gene in this region has been shown to play a role in cell proliferation and body size in cattle and sheep (Mastranestasis et al. 2016; Gonzalez et al. 2020).

Among the genes within ±500 kb from the significant SNP on ECA22, the *RBL1*, *SRC* and *SAMHD1* genes were previously detected among overrepresented genes in a GO term analysis for show jumping SWB horses (Ablondi, Eriksson, et al. 2019). All three genes were identified as a potential candidate genes associated with mentality and postsynaptic signalling in show jumping SWB horses (Ablondi, Eriksson, et al. 2019). In addition to this, SRC has a role in osteoporosis in mice due to non‐functional osteoclasts (Lowe et al. 1993).

Among other genes in the window on ECA22, the *SOGA1* gene encodes a protein that reduce glucose production and plays a role in autophagy (Cowerd et al. 2010). Other detected genes were *GHRH*, known for its role in growth in human and animals, such as carcass traits in Korean cattle (Cheong et al. 2006), and *CTNNBL1* that is associated with body weight and height in humans (Andreasen et al. 2009), and muscle formation and body weight in cattle (Mancini et al. 2014).

An association of these genes with L.walk is not clear, except that the growth and conformation of the horse, for example, length of legs, may influence aspects like stride length. There are also other nearby genes in ECA22, like *NNAT*, that is involved in brain and nervous system development in humans (Safran et al. 2021), that could potentially influence gaits.

The top SNP for the factor L.trot.hind was located nearby three genes on ECA15. One of these was the *NCK2* gene that has an impact on preosteoblastic and osteoblastic migration and bone mass in human by bone formation activity (Aryal and Noda 2015). As the L.trot.hind factor is related to hind leg position and activity, as well as elasticity during trot, it is possible that skeletal development influencing angles, and/or the area of muscle attachment, could be of importance for this trait.

### 4.6 Additional Remarks

These findings need to be validated in future studies, and the function of the potential candidate genes related to the phenotypes remains to be clarified. The regions with SNPs associated with factors related to body height are well known from numerous previous studies in different species, which shows that the data quality and methods applied were sufficient to detect genomic regions of clear importance for the traits. Even for height at withers, the full genetic background of height in horses remains to be revealed. It should also be noted that besides protein coding genes, also, for example, regulatory regions could be of importance for the studied traits. Besides the value of learning more about the biology behind desired phenotypes, the results may also be of interest for future assessment and selection of horses. If, for example, a desired trait, such as an arched neck in SWB would be genetically associated with risk factors for developing laminitis, then that would need to be considered in the breeding goal.

## 5 Conclusions

By the use of single‐step genome‐wide association analysis of factors for evaluated and linearly scored young horse test traits in SWB horses, we detected some plausible candidate regions and genes of interest to study further. These included a well‐known region on ECA3 for factors related to body size, as well as more novel candidate genes for factors related to jumping and to the shape and position of the neck, both for which the detected top SNPs were found within genes.

## Author Contributions

Susanne Eriksson, Sofia Mikko and Åsa Gelinder Viklund initiated the study. Susanne Eriksson and W. Freddy Fikse designed and supervised the study. Åsa Gelinder Viklund provided pre‐edited data and trait information. Sofia Mikko was main applicant for the funding of the genotyping. Anahit Nazari‐Ghadikolaei carried out the data analysis with contributions from W. Freddy Fikse, Susanne Eriksson and Anahit Nazari‐Ghadikolaei drafted the manuscript. Sofia Mikko scrutinised the detected genomic regions. All authors read, edited and approved the final manuscript.

## Ethics Statement

Not applicable as the hair samples from Swedish Warmblood horses were originally collected for parentage testing and stored in the biobank at the Animal Genetics Laboratory, SLU. The Swedish Warmblood Association approved the samples to be used in this research. No additional samples were collected for this study.

## Conflicts of Interest

The Swedish Warmblood Association has provided the phenotype and pedigree data for this study, and Åsa Gelinder Viklund has regular commitments to Swedish Warmblood Association, regarding the routine genetic evaluation. We declare that there are no other conflicts of interest.

## Supporting information


Figure S1.

Table S1.

Table S2.

Table S3.

## Data Availability

Restrictions apply to the availability of these data, which were used under licence for this study. Data are available from the authors with the permission of the Swedish Warmblood Association.
