# Supplementary material for: Single‐Step Genome‐Wide Association Study of Factors for Evaluated and Linearly Scored Traits in Swedish Warmblood Horses
Source: J Anim Breed Genet. 2025 Jan 4;142(5):499–512. doi: 10.1111/jbg.12923 (PMC12340361; doi:10.1111/jbg.12923)
Supplement: Supplementary file 1 — Figure S1. [file JBG-142-499-s003.docx]

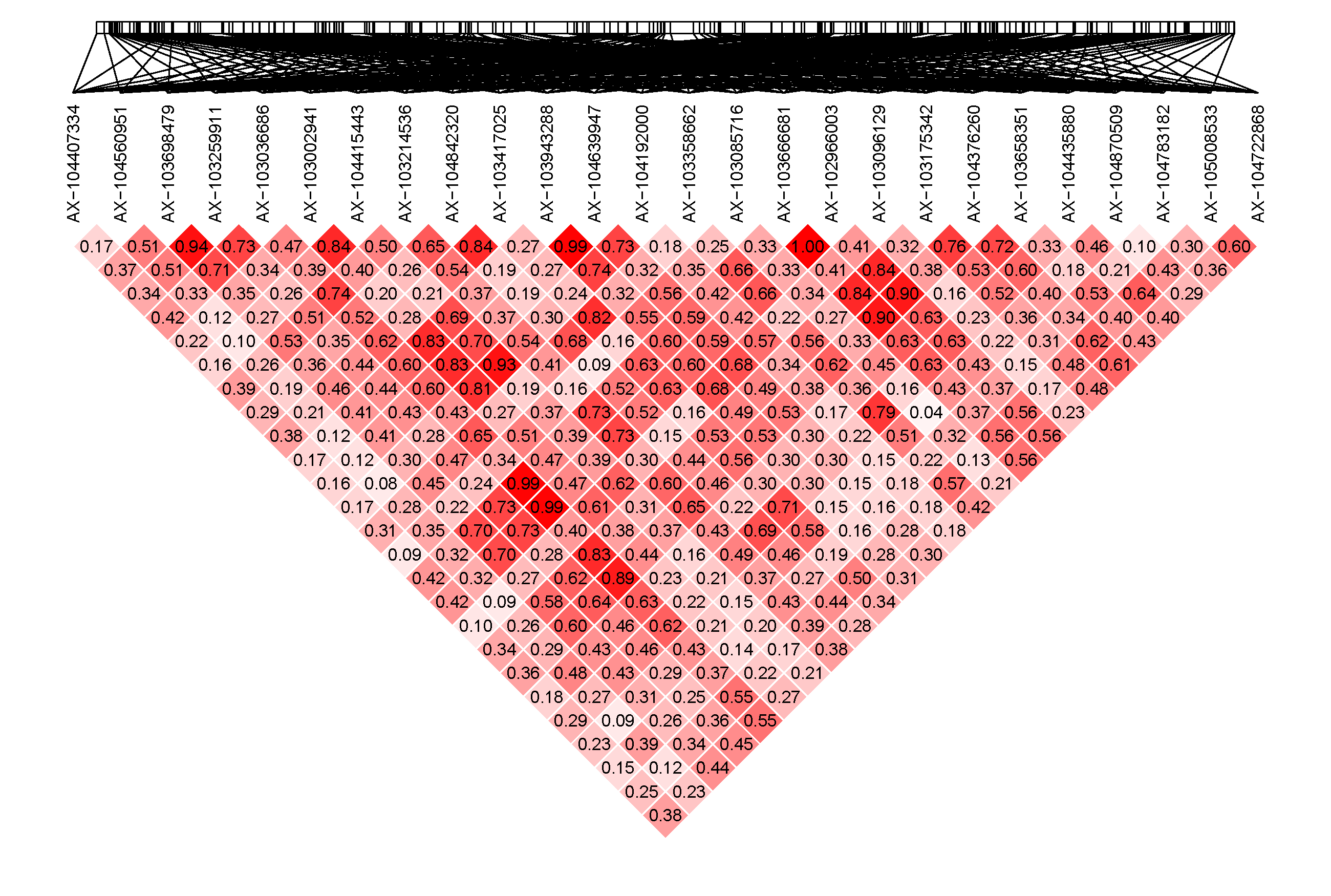


**a**


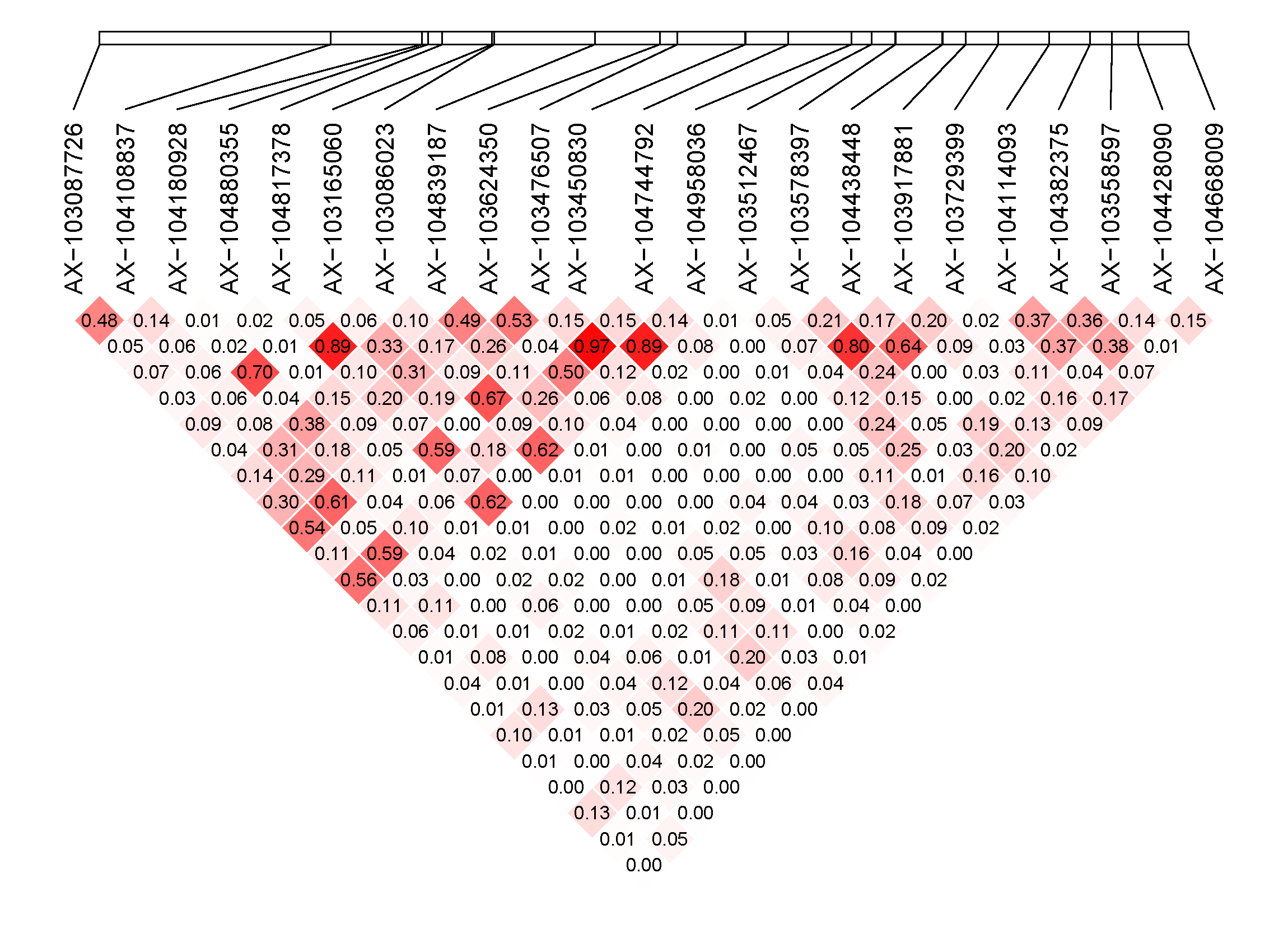


**b**


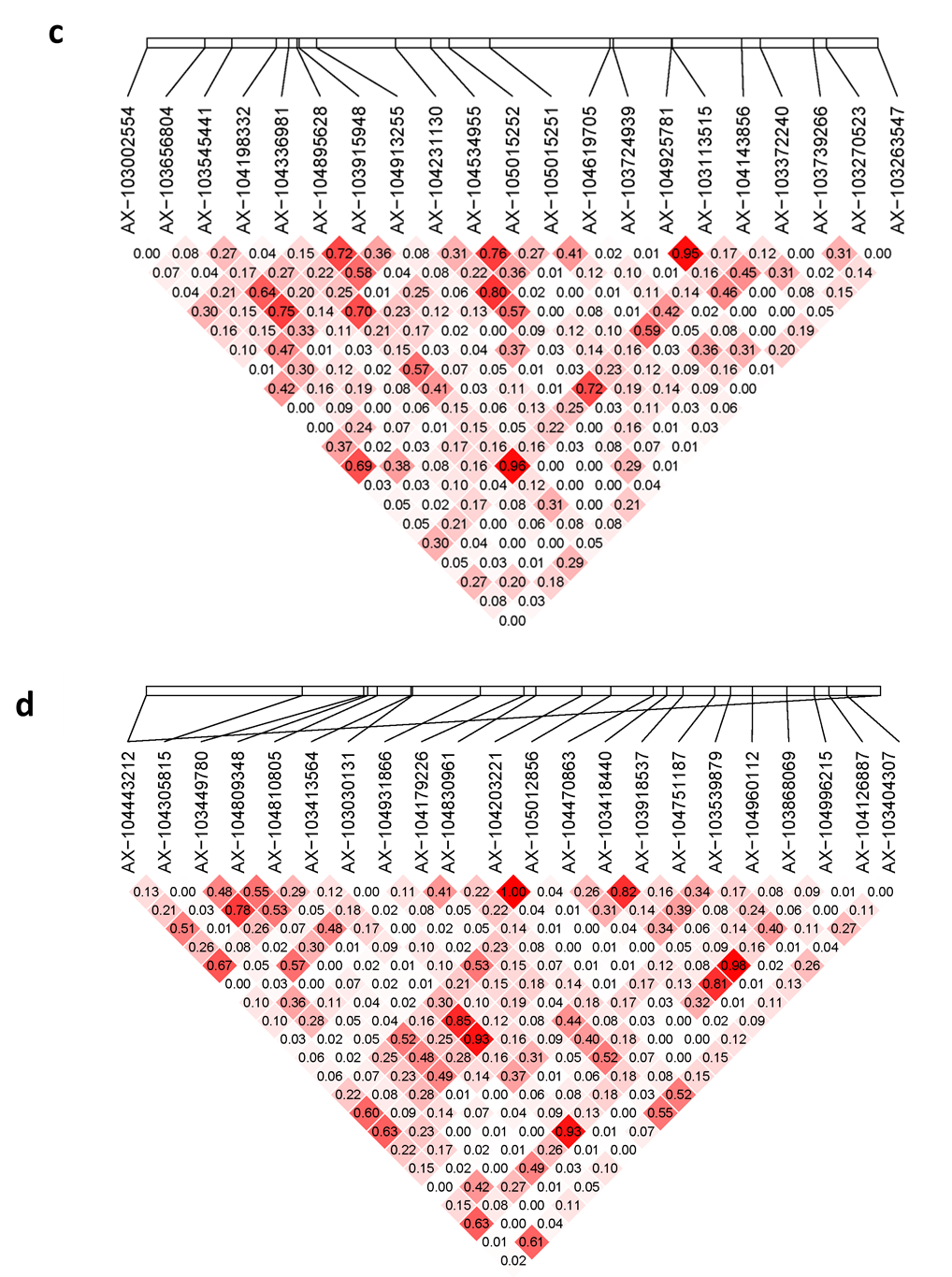


**Supplementary Figure 1**. Linkage disequilibrium (LD) between significant SNPs for factors a) related to body height and size: E.size and L.height and L.type, b) neck conformation (L.neck), c) jumping traits (E.jump) and d) walk traits (L. walk), in SWB horses. Top SNPs are shown in blue squares. The SNPs marked in a) (AX-104376260 and AX-103658351) were significantly associated with more than one factor related to body size, height and/or type.
