# Supplementary material for: Single‐Step Genome‐Wide Association Study of Factors for Evaluated and Linearly Scored Traits in Swedish Warmblood Horses
Source: J Anim Breed Genet. 2025 Jan 4;142(5):499–512. doi: 10.1111/jbg.12923 (PMC12340361; doi:10.1111/jbg.12923)
Supplement: Supplementary file 2 — Table S1. [file JBG-142-499-s004.docx]

Supplementary Table 1. Descriptive parameters and estimated genetic (σ^2^_a_) and residual (σ^2^_e_) variances and heritability (h^2^) with standard errors as subscripts for evaluated (N=20,935) and linearly scored (N=6436) factors used for ssGWAS in SWB horses.

| **Factor** | **Higher values corresponds to** | **Mean** | **SD** | **σ^2^_a_** | | **σ^2^_e_** | | **h^2^** | |
| --- | --- | --- | --- | --- | --- | --- | --- | --- | --- |
| *Evaluated* | | | | | | | | | |
| E.jump | Better jumping ability | 0.19E-05 | 0.954 | 0.247 | _0.015_ | 0.502 | _0.012_ | 0.32 | _0.018_ |
| E.gaits | Better gait quality | -0.37E-05 | 0.807 | 0.291 | _0.012_ | 0.237 | _0.008_ | 0.53 | _0.018_ |
| E.size | Taller with better conformation | 0.96E-04 | 0.774 | 0.372 | _0.016_ | 0.242 | _0.010_ | 0.59 | _0.020_ |
| E.conf | Better conformation | -0.59E-04 | 0.612 | 0.116 | _0.007_ | 0.242 | _0.006_ | 0.29 | _0.017_ |
| *Linearly scored* | | | | | | | | | |
| L.jump | Weaker, slower, more forward takeoff, less bent forelegs, less rounded back, tighter haunches, less scope, stiffer, less careful, slower reaction, less balanced, less secure distance estimation | -0.24E-03 | 0.903 | 0.280 | _0.026_ | 0.370 | _0.020_ | 0.33 | _0.023_ |
| L.quick | Slower takeoff and reaction | -0.50E-02 | 0.834 | 0.090 | _0.017_ | 0.548 | _0.017_ | 0.13 | _0.025_ |
| L.behavior | More tense and unfocused, less secure distance estimation | -0.99E-02 | 0.863 | 0.047 | _0.013_ | 0.668 | _0.017_ | 0.06 | _0.017_ |
| L.walk | Stiffer, more uneven walk with shorter stride length | -0.38E-03 | 0.923 | 0.200 | _0.026_ | 0.552 | _0.023_ | 0.24 | _0.030_ |
| L.trot | Less elastic trot with shorter stride length, less foreleg activity | 0.62E-02 | 0.831 | 0.204 | _0.024_ | 0.411 | _0.020_ | 0.27 | _0.025_ |
| L.trot_hind | Less elastic trot with less active hind legs positioned more behind the body | -0.12E-02 | 0.798 | 0.136 | _0.017_ | 0.363 | _0.015_ | 0.21 | _0.026_ |
| L.canter | Less even, less balanced, flatter, and less elastic canter with shorter stride length and more downhill direction of movements | 0.19E-02 | 0.878 | 0.235 | _0.029_ | 0.494 | _0.024_ | 0.31 | _0.028_ |
| L.direction | More downhill body and gait direction, less balanced canter | -0.89E-03 | 0.721 | 0.098 | _0.015_ | 0.389 | _0.014_ | 0.14 | _0.023_ |
| L.correct | Less toed-out or more toed-in forelegs with more paddling or less winging movements | 0.12E-02 | 0.799 | 0.098 | _0.018_ | 0.506 | _0.017_ | 0.14 | _0.027_ |
| L.height | Lower height at withers, more short legged, lower withers | -0.29E-03 | 0.839 | 0.269 | _0.029_ | 0.381 | _0.023_ | 0.51 | _0.028_ |
| L.length | Shorter body, neck and loins | -0.17E-02 | 0.901 | 0.160 | _0.024_ | 0.630 | _0.023_ | 0.21 | _0.030_ |
| L.type | Taller and heavier body type | -0.68E-03 | 0.705 | 0.175 | _0.024_ | 0.422 | _0.021_ | 0.35 | _0.037_ |
| L.neck | Steeper shoulder, more horizontal position of neck and straighter neck shape | -0.30E-03 | 0.828 | 0.229 | _0.026_ | 0.389 | _0.021_ | 0.34 | _0.033_ |
